# Whole-body vibration training in obese subjects: A systematic review

**DOI:** 10.1371/journal.pone.0202866

**Published:** 2018-09-05

**Authors:** Matteo Zago, Paolo Capodaglio, Cristina Ferrario, Marco Tarabini, Manuela Galli

**Affiliations:** 1 Dipartimento di Elettronica, Informazione e Bioingegneria, Politecnico di Milano, Milano–Italy; 2 Fondazione Istituto Farmacologico “Filippo Serpero”, Milano–Italy; 3 Research Laboratory in Biomechanics and Rehabilitation, Orthopedic Rehabilitation Unit, IRCCS Istituto Auxologico Italiano, Ospedale San Giuseppe, Piancavallo (VCO), Italy; 4 Dipartimento di Meccanica, Politecnico di Milano, Milano–Italy; Berner Fachhochschule, SWITZERLAND

## Abstract

**Objective:**

(i) to determine the outcomes of whole-body vibration training (WBVT) on obese individuals, and the intervention settings producing such effects; (ii) identify potential improper or harmful use of WBVT.

**Design:**

Systematic review.

**Data sources:**

Medline, Scopus, Web of Science, PEDro and Scielo until July 2018.

**Eligibility criteria:**

Full papers evaluating the effect of WBVT on body composition, cardiovascular status and functional performance in obese adults. Papers with PEDro score<4 were excluded.

**Study appraisal and synthesis:**

Risk of bias and quality of WBVT reporting were assessed with PEDro scale (randomized controlled trials) or TREND checklist (non-randomized studies) and a 14-items checklist, respectively. Weighted acceleration, daily exposure and Hedges’ adjusted g were computed.

**Results:**

We included 18 papers published 2010–2017. Typical interventions consisted in three sessions/week of exercises (squats, calf-raises) performed on platforms vibrating at 25–40 Hz (amplitude: 1–2 mm); according to ISO 2631–1:1997, daily exposure was “unsafe” in 7/18 studies.

Interventions lasting ≥6 weeks improved cardiac autonomic function and reduced central/peripheral arterial stiffness in obese women; 10 weeks of WBVT produced significant weight/fat mass reduction, leg strength improvements as resistance training, and enhanced glucose regulation when added to hypocaloric diet. No paper evidenced losses of lean mass. Isolated cases of adverse effects were reported.

**Summary:**

To date, WBVT is a promising adjuvant intervention therapy for obese women; long-term studies involving larger cohorts and male participants are required to demonstrate the associated safety and health benefits. The therapeutic use of WBVT in the management of obese patients is still not standardised and should be supported by an extensive knowledge on the causality between vibration parameters and outcomes.

## Introduction

Obesity is a severe and growing health problem which increases the risk of debilitating and death-leading diseases [1,2]. In obese individuals, hypertriglyceridemia and insulin resistance lead to impaired fasting glucose, high blood sugar levels, inflammation and visceral adipose tissue (VAT) accumulation [3]. These elements contribute to several adverse cardiovascular outcomes, partly due to cardiac autonomic dysfunction [4–7]: hypertension, increased sympathovagal balance and arterial stiffness, reduced heart rate (HR) variability, endothelial dysfunction, and eventually enhanced risk of coronary heart disease, stroke and cardiovascular death [3,4,8,9]. Cardiovascular complications are often associated with leg sarcopenia (loss of muscle mass), with consequent postural instability and increased risk of falls [10–12]. Menopause and aging additionally impact upon the health of obese individuals, aggravating cardiovascular status [13–15], VAT accumulation, sarcopenia, and reducing bone mineral density [16].

In obese individuals, even a modest weight loss (5–10% of body weight) helps to alleviate cardiovascular risk [3]. Thus, weight and VAT loss are primary treatment goals, conventionally achieved through dietary modifications [3,17,18], behavioural correction and/or exercise prescription [19–21]. However, the success rate of therapy for obesity is very low: dieting may work in the short term, but severe dietary restriction alone reduces muscle mass and leads to a decline in physical fitness [22]; traditional exercise, such as aerobic and resistance training, improves heart rate variability, physical strength and body composition [23,24]. However, the majority of obese people maintains a sedentary lifestyle and is reluctant to enrol and persist in conventional exercise programs due to physical limitations, musculoskeletal discomfort and lack of self-motivation [25].

In the last two decades, whole-body vibration training (WBVT) emerged as an alternative exercise modality for strength training [26–28]. WBVT involves exercising on a vibrating platform. Vibrations mechanically generate rapid variations in the length of the muscle-tendon complex [29], stimulating repetitive eccentric-concentric muscular work and reflexive muscle contractions [30,31]. WBVT was first recognized as an alternative to resistance exercise for its ability in enhancing force and power in skeletal muscle [28,32]. Evidence that body vibrations slow down fat accumulation and reduce adipogenesis in rats [33,34] suggested a possible clinical use of WBVT in the treatment of obesity. Indeed, WBVT improved body composition [35,36], muscle strength [28] and cardiovascular function in various populations, including obese individuals [37,38].

To date no systematic review summarised the outcomes of WBVT on obese subjects: although WBVT is gaining growing interest as an exercise prescription for obese patients, there is no clear consensus about the vibration exposure (i.e. amplitude, frequency, duration), and exercise performed on the platform to obtain positive effects and avoid over-training or injuries [39]. Therefore, this systematic review aimed at (i) defining the outcomes of WBVT on obese individuals and which combination of vibration and exercise setting enables to attain such effects, and (ii) identifying gaps of knowledge that may lead to improper use of WBVT with consequent harmful effects.

## Methods

This systematic review was undertaken using the preferred reporting guidelines for systematic reviews and meta-analysis (PRISMA) [40].

### Search strategy

A systematic literature search was performed in July 2018 on the following electronic databases (from 1990): Web of Science, PubMed MEDLINE, Scopus, Mendeley, PEDro, Scielo. Customised queries including keywords and Boolean logic with AND/OR operators were entered in the search engines in this form: (“whole-body vibration” OR “vibrating platforms” OR “vibration training”) AND (“obese” OR “obesity”), with document type set to “Article”.

The search was limited to full original articles written in English and to investigations on human subjects. Bibliographies of identified papers were hand searched for supplemental relevant items.

### Eligibility criteria and study selection

Randomised control trials, quasi-experimental studies and observational case series were included. The PICO (P = Patients, I = Intervention, C = Comparison, O = Outcomes) method was used to define inclusion criteria: P = obese adults (age≥18 years) with body mass index (BMI)≥30 kg∙m^-2^; studies including among them few pre-obese participants were included if the total sample BMI was on average ≥30 kg∙m^-2^; studies assessing obese patients with documented comorbidities (i.e. Diabetes Mellitus) were also included; I = whole-body vibration training; C = (1) comparison between pre- and post-intervention, (2) comparison with no whole-body vibration under the same exercise condition, or with other forms of physical activity/intervention; O = body composition and cardiovascular status (primary outcomes), and/or measures of biomechanics/functional performance (secondary outcomes).

We excluded studies not primarily focused on the evaluation of WBVT, studies assessing the acute effects during WBVT, animal studies, studies including overweight (not obese) or non-adult participants, studies that used focal rather than whole-body vibration. Two reviewers (CF and MZ) independently screened titles and abstracts of the identified records and took decision about items retention in a blinded manner. Any disagreement between the examiners was resolved by a technical discussion involving a third reviewer (MT).

### Methodological quality

The Physiotherapy Evidence Database (PEDro) scale [41] was used to evaluate whether the selected randomized controlled trials were scientifically sound (9–10 = excellent, 6–8 = good, 4–5 = fair, and <4 = poor). When the score was unavailable on the PEDro database, articles were rated independently by two researchers (MZ and CF, S1 Table). Papers with poor PEDro score were excluded. The quality of non-randomized (quasi-experimental and case series) studies was assessed with the Transparent Reporting of Evaluations with Nonrandomized Designs (TREND) checklist [42].

The quality of whole-body vibration reporting was assessed using the checklist of the International Society of Musculoskeletal and Neuronal Interactions [43], consisting of 13 items regarding the vibration protocol plus an item addressing the supervision received during WBVT [44] (S3 Table).

### Data synthesis and analysis

A standardised data extraction and appraisal form was developed to collect the key features of each study: authors/year, study design, demographics of participants to WBVT, body composition (BMI), status and comorbidities, intervention details (vibration frequency, acceleration and displacement, duration and nature of performed exercises), main outcomes of WBVT grouped in the following domains: (i) body composition, (ii) cardiovascular parameters and (iii) hormonal concentrations, strength and functional changes. For each study, the weighted acceleration was derived by multiplying the amplitude of the vibration by the coefficient (at the stimulus frequency) of the weighting curve, and the daily exposure was computed according as per ISO 2631–1:1997, Annex B [45].

After reviewing the results of the selected studies, it was decided that a meta-analysis was not appropriate because the treatment protocols and the outcomes measures substantially varied across studies. To estimate the effect size of interventions yielding significant results, Hedges’ adjusted g was computed based on the data provided in the examined articles; small, medium and large effects corresponded to g = 0.2, g = 0.5 and g = 0.8, respectively [46].

## Results

### Literature search results

**Fig 1:**
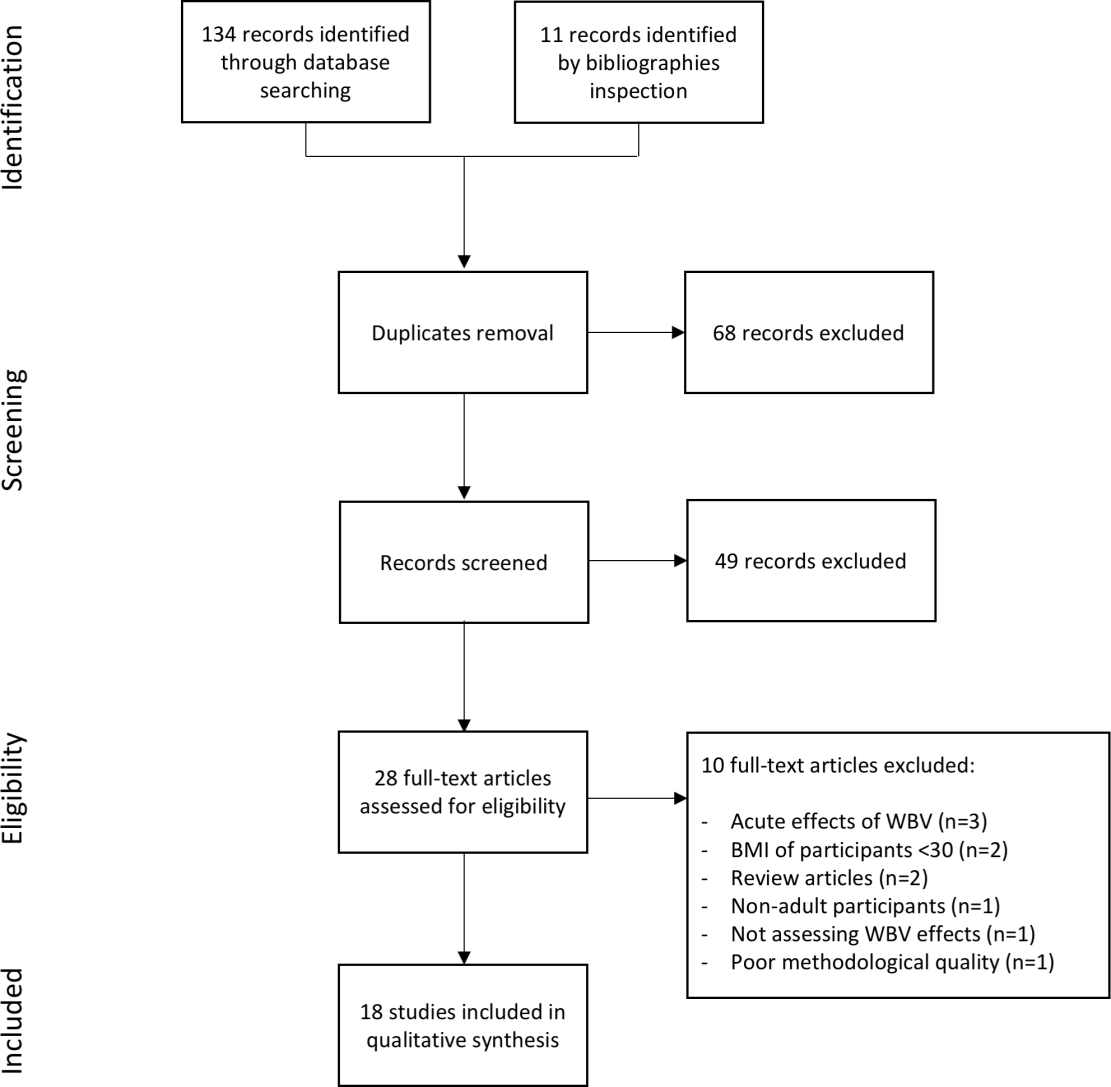
PRISMA diagram of study selection.

A total of 134 records were retrieved from the electronic databases. Eleven items were added inspecting reference lists and review articles. After removing 68 duplicates, titles and abstracts screening led to exclude 49 papers. Out of the remaining 28 articles, 10 failed to meet inclusion criteria. The main reasons for exclusion were: studies assessing the acute effects of WBVT or studies on participants with BMI<30 kg∙m^-2^; one paper was excluded because of poor methodological quality (PEDro score<4). Eighteen papers published from 2010 to 2017 were included in the review [26,47–63]: 16 were randomized control trials, one a quasi-experimental study [60] and one an observational case series study [54]. The selection process is summarised in Fig 1.

### Description of the included studies

#### Study population

The sample size of obese participants ranged from n = 7 to n = 40 (mean age range: 20 to 59 years). A total of 321 subjects were involved, but we could not exclude subjects overlapping in studies conducted by the same research group. Sixteen out of 18 papers focused on obese women (Table 1); six of them investigated the effect of WBVT after menopause [26,49,50,52,57,59]. Four studies assessed a small male sample (n = 4–10 *[45,48,52,53]*). The majority of studies involved obese participants with no further pathologies, apart from [62] and [61] who included patients diagnosed with type-2 Diabetes Mellitus and fibromyalgia, respectively; Wong et al. [49] and Figueroa et al. [48] included prehypertensive and hypertensive women with brachial systolic blood pressure (BP) higher than 120 mmHg.

**Table 1:** Quality of the examined papers and demographics of participants subject to whole body-vibration training (WBVT), combined with other treatment, if appropriate.

Author and reference	Year	PEDro score (0–10)	WBV score (0–14)	WBV sample size (females)	Age (years)	BMI (kg∙m^-2^)	Status / comorbidities
Vissers et al. [58]	2010	4	6	18 (9)	43.3±9.6	30.8±3.4	Healthy
Figueroa et al. [56]	2012	5	9	10 (10)	21.0±2.0	29.9±0.8	Healthy, sedentary
Miyaki et al. [60]	2012	4	7	12 (12)	42.0±2.0	32.0±1.0	Healthy
Wilms et al. [51]	2012	4	9	7 (7)	43.1±3.5	37.4±1.3	Healthy
Adsuar et al. [61]	2013	7	7	18 (18)	53.0±1.2	29.6±4.2	Diagnosed with Fibromyalgia
Sañudo et al. [62]	2013	5	7	20 (10)	72.0±8.0	31.0±6.9	Type 2 Diabetes Mellitus
Milanese et al. [53]	2013	4	5	13 (13)	46.8±7.8	5.7±0.7	Healthy
Zaki [57]	2014	7	6	40 (40)	57.3±5.3	35.5±6.5	Healthy, postmenopausal
Bellia et al. [63]	2014	6	8	12 (8)	42±4.0	33.1±2.8	Healthy
Figueroa et al. [26]	2014	5	8	15 (15)	56.0±3.0	32.8±3.6	Healthy, postmenopausal
So et al. [54]	2014	5	6	16 (16)	43.3±5.5	31.2±4.0	Healthy, sedentary
Figueroa et al. [48]	2014	6	6	WBV-n: 12 (12) WBV-h: 12 (12)	WBV-n: 58.0±1.0 WBV-h: 56.0±1.0	WBV-n: 34.6±0.9 WBV-h: 33.7±1.5	Prehypertension (WBV-n) or stage 1 (WBV-h) hypertension, sedentary
Figueroa et al. [59]	2015	7	8	14 (14) WBV+L: 13 (13)	58.0±1.0 WBV+L: 58.0±1.0	35.0±0.9 WBV+L: 33.8±1.1	Healthy, postmenopausal, sedentary WBV+L: L-Citrulline supplementation, 6 g/day
Wong et al. [50]	2016	6	8	14 (14) WBV+L: 13 (13)	58.0±4.0 WBV+L: 58.0±4.0	35.0±3.4 WBV+L: 32.7±3.1	Healthy, postmenopausal, sedentary WBV+L: L-Citrulline supplementation, 6 g/day
Wong et al. [49]	2016	4	10	12 (12)	59.0±1.0	33.7±1.2	Postmenopausal, stage 1 hypertension, sedentary
Severino et al. [52]	2016	5	8	13 (13)	58.0±1.0	34.6±1.3	Healthy, postmenopausal, sedentary
Yang et al. [47]	2017	6	6	12 (4)	26.0±7.3	34.4±1.9	Healthy
Alvarez-Alvarado et al. [55]	2017	6	9	25 (25)	20.0±1.0	30.7±0.7	Healthy

Control groups included non-exercising, matched participants in seven papers [26,48,49,52,53,55,61], women undergoing resistance training [57], hypocaloric diet [63], and a general exercise program [51], or subject to diet restrictions combined with aerobic training [54]; two studies investigated the effects of a combination of WBVT and L-Citrulline supplementation [50,59].

#### Intervention

WBVT mainly consisted in a series of exercises performed on the platform, namely squats (at different degrees of knee flexion) and calf-raises (Table 2); two studies prescribed static upright standing sessions with a knee flexion angle ranging from 20 to 45 degrees [47,61]. Interventions lasted 6 to 12 weeks, with a frequency of three sessions per week in 13/18 studies. A single trial evaluated the effect of a 24-week intervention and related long-term effects [58]. Platforms used applied synchronous vertical vibrations in 9/18 studies and side-alternating (or rotating) vibrations in 2/18 studies; seven studies did not provide vibration type. Vibration frequency ranged from 12.5 to 60 Hz, while the most common choice was between 25 and 40 Hz, with a peak-to-peak displacement (amplitude) of 1–2 mm. Exercises bouts lasted 30 to 60 s, with a work:rest ratio from 1:1 to 1:2. According to ISO 2631–1:1997, daily exposure was “tolerable” in 5/18 studies, “unsafe” in 7/18 studies and not available due to lack of data in the remaining studies.

**Table 2:** Whole-body vibration training details (VV: Vertical vibration; RV: Rotating vibration; NA: Not available). Weighted exposure was compared with ISO 2631–1:1997 [45] boundaries.

Reference	Frequency (Hz) and type of vibration	Peak-to-peak displacement (mm), peak acceleration (g)	Weighted vibration (m∙s^-2^) and exposure	Posture and exercises performed on the plate	Number of repetitions x exercise time + rest period, intervention frequency and duration
[58]	30 to 40, VV	NA	Unpredictable	Static, then dynamic exercises like squatting, deep squatting, calf-raises, lunges, curl-ups, push-ups.	(10 to 22) x (30 to 60) s + (30 to 60) s rest, NA/week for 24 weeks
[56]	25 to 30, NA	1 to 2 mm, 2.83 to 4.86 g	19.4 m∙s^-2^, unsafe	Static and dynamic semi-squats (60° knee flexion), wide-stand semi-squats and calf-raises. External load (5–10% body weight) in the last two weeks.	(30 to 60) s per exercise + (30 to 60) s rest, 3/week for 6 weeks
[60]	30–35, VV	2 mm	12.7 m∙s^-2^, unsafe	High squats; deep squats; wide-stance squats and lunges.	30 x 30 s + 30 s rest for 30 min, 3/week for 12 weeks
[51]	30, VV	2 mm	12.7 m∙s^-2^, unsafe	Week 1: lunges, biceps curls and shoulder relaxation exercises. These were complemented in week 2 by exercises for the sural muscle and one leg stands, in week 3 by exercises for the abdominal side muscles, triceps curls and side crunches and in week 4 by press-ups, exercises for the lower abdominal muscles and the pelvis muscles.	(5 to 16) x 30 s + 30 s rest, 3/week for 6 weeks
[61]	12.5, RV	NA	Unpredictable	Standing, 45° of knee flexion.	6 x (30 to 45) s + 60 s rest, 3/week for 12 weeks
[62]	12 to 16, NA	4 mm	36.0 m∙s^-2^, unsafe	8 dynamic and static exercises: lunge, step up and down, squat, calf raises, pivot, shoulder abduction with elastic bands, shoulder abduction with elastic bands while squatting, arm swinging with elastic bands.	8 exercises x (30 to 60) s + 30 s rest, 3/week for 12 weeks
[53]	40 to 60, VV	2 to 5 mm	4 m∙s^-2^, tolerable	20 sequential unloaded static leg and arm exercises	20 x (30 to 60) s + 15 s rest, 2/week for 10 weeks
[57]	16, RV	NA	Unpredictable	NA	(3 to 10) x 60 s + 60 s rest, 3/week fr 8 weeks
[63]	30, VV	2 mm	12.7 m∙s^-2^, unsafe	Squat, 70° knee flexion	10 x 60 s +60 s rest, 3/week for 8 weeks
[26]	25–30, NA	1 mm	5.1 m∙s^-2^ tolerable	Dynamic and static semi-squats and lunges with a 120° knee flexion angle, squats with a 90° knee flexion angle and calf-raises.	(1 to 2 exercise set) x (30 to 45) s x 60 s rest, 3/week for 6 weeks
[54]	30–35, NA	NA	Unpredictable	Squats, wide-stance squats, deep squats, lunges, push-ups, triceps dips, and front plank.	(12 to 24 exercises) x 30 s + 30 s rest, 3/week for 12 weeks
[48]	25 to 40, NA	1 to 2 mm	5.1 m∙s^-2^, tolerable	Squats with a 90° and 120° knee flexion angle, wide-stand semi-squats, and calf raises.	(1 to 6) x (30 to 60) s + (30 to 60) s rest, 3/week for 12 weeks
[59]	25 to 40, VV	1 to 2 mm	5.1 m∙s^-2^, tolerable	Squat, normal stance, 90° and 120° of knee flexion; squat, wide-stance, 120° of knee flexion; calf-raises.	(1 to 5) x (30 to 60) s + (30 to 60) rest, 3/week for 8 weeks
[50]	25 to 40, NA	1 to 2 mm	5.1 m∙s^-2^, tolerable	Squat, normal stance, 90° and 120° of knee flexion; squat, wide-stance, 120° of knee flexion; calf-raises.	(1 to 5) x (30 to 60) s + (30 to 60) rest, 3/week for 8 weeks
[49]	25 to 40, VV	4.3 to 21.3 g	42.0 m∙s^-2^, unsafe	Squats at 90° and 120° knee flexion angle with normal stance, squat at 120° knee angle with wide stance, calf raises with maximal heel elevation.	(1 to 5) x (30 to 60) s + (30 to 60) s rest, NA/week for 8 weeks
[52]	25 to 40, NA	1 to 2 mm	5.1 m∙s^-2^, tolerable	Squats at 90° and 120° knee flexion angles (normal stance), squat at 120° knee (wide-stance); calf raises with maximal plantar flexion.	(3 to 7) x (30 to 60) s + 30 to 60 s, NA/week for 8 weeks
[47]	25, VV	3.9 mm	31.2 m∙s^-2^, unsafe	Standing, 20° of knee flexion, upright trunk.	5 x 60 s + 60 s rest, 3/week for 6 weeks
[55]	30 to 35, VV	NA	Unpredictable	Squats at a 90° knee flexion angle, semi-squats at 120° knee angle, wide-squat at 90° knee angle and calf-raises.	(2 to 8) x exercise, (30 to 60) s + (60 to 45) s rest, NA/week for 6 weeks

### Quality of the included studies

Of the 16 included RCT papers, 9 were deemed with “fair”, and 7 with “good” methodological quality according to the PEDro score. Eligibility (item 1 in the PEDro scale and item 3 in the TREND checklist) was fulfilled by 12/18 papers (S1 and S2 Tables); all papers presented between-groups comparison and point/variability estimates (items 10–11 in the PEDro scale and item 17 in the TREND checklist); 16 papers randomly allocated subjects; 17 papers reported similar demographics and anthropometrics at baseline. Rather, only few studies could dispose of blind assessors.

The quality of WBVT reporting reached 10/14 once [49] and was on average 7.5/14 (S3 Table). All selected studies explicitly reported platform model and vibration frequency, but few papers detailed peak acceleration (n = 2), accuracy of vibration parameters (n = 0), footwear (n = 0) and support devices (n = 5).

### Summary of evidence

A wide spectrum of outcomes was assessed (Table 3). Thirteen papers evaluated body composition parameters. Body composition measurement was performed with dual-energy X-ray absorptiometry by eight studies [48,50,53,54,57,59,60,63], bioelectrical impedance by three studies [51,58,62] and air-displacement plethysmography by a single study [52]. Participants’ body weight decreased after treatment in eight studies [53,57,58,60–63] and remained unchanged in five [26,50–52,59]; BMI and fat mass decreased more in WBVT patients than in controls exposed to aerobic training and dietary restriction [51]. Weight loss varied from 5% (~1 kg, small effect) [53,58,61] to 10% (large effect) [54,60]. Similarly, fat mass reduced from 2% to 6% (small to large effects) in seven studies [51–54,59,60,62] and did not change in [26]; Bellia et al. found a higher reduction in fat mass after WBVT than in participants undergoing hypocaloric diet [63]. No paper evidenced an adverse loss of lean mass. Conversely, an increase of lean mass was observed by Miyaki et al. [60]; Figueroa et al. [59] found that leg lean mass increased by 2% following WBVT and L-citrulline supplementation for 6 weeks. VAT area modification was measured in two studies reporting very small [58] to very large [54] effects (about 10 and 50 cm^2^, respectively). Waist circumference decreased by 2–10 cm (medium to large effects) in four studies [51,53,57,60]. Body impedance analysis revealed an enhancing effect of WBVT on the bio-electrical phase angle [51], as compared to controls undergoing general strength exercises. Hip and lumbar spine bone mineral density increased with a small effect up to 0.05 g∙cm^-2^ after 8–10 weeks of WBVT [53], similarly to resistance exercise [57].

**Table 3:** Outcomes of whole-body vibration training. Hedges adjusted g effects size and statistical significance were reported for each variable.

	Domain		
Reference	Body composition	Cardiovascular parameters	Hormonal, hematic and functional parameters
[58]	BW decreased (g = 0.05, p<0.05) and was maintained in the long-term (g = 0.53, p<0.05); visceral adipose tissue decreased (g = 0.01, p<0.05).		
[56]		Systemic arterial stiffness decreased: systolic aortic BP decreased (g = 1.96 and g = 2.24, p<0.05). Sympathovagal balance improved: total power increased (g = 2.60, p<0.05), Low Frequency decreased (g = 3.45). HR decreased (g = 5.00, p<0.05).	
[60]	BW (g = 2.34, p<0.05), % fat mass (g = 1.8, p<0.05), waist circumference (g = 3.92, p<0.05) decreased; % lean body mass increased (g = 0.74, p<0.05).	Systolic (g = 2.43, p<0.05) and diastolic (g = 2.35, p<0.05) BP, mean arterial pressure (g = 2.55, p<0.05), HR decreased (g = 3.16, p<0.05). Carotid-femoral (g = 2.62, p<0.05) and brachial-ankle (g = 2.25, p<0.05) PWV decreased.	Triglycerides (g = 2.22, p<0.05), total (g = 3.41, p<0.05) and LDL cholesterol decreased (g = 2.83, p<0.05); Peak oxygen uptake increased (g = 4.12, p<0.05).
[51]	BW did not change (g = 0.04, p<0.05); waist circumference (g = 0.83, p<0.05) and % fat mass decreased (g = 0.50, p<0.05). Phase angle enhanced (g = 0.67, p<0.05).		Resting energy expenditure increased (g = 0.87, p<0.05).
[61]	BW decreased (g = 0.04, p<0.05).		
[62]	BW (g = 0.16, p<0.05), waist circumference (g = 0.48, p<0.05), waist to hip ratio (g = 0.65, p<0.05), % of body fat (g = 0.24, p<0.05) reduced.	Blood flow velocity increased (g = 0.32, p<0.05), maximum diastolic velocity (g = 0.83, p<0.05) and pulsatility index (g = 0.06, p<0.05) decreased.	
[53]	BW (g = 0.15, p = 0.033), total body (g = 0.17, p = 0.033) and trunk (g = 0.86, p = 0.004) fat mass reduced; body circumferences but the wrist decreased (g~1, p<0.01). Bone mineral density slightly increased (g = 0, p<0.001).		Strength increase: leg press (g = 3.31, p<0.001), leg extension (g = 5.29, p = 0.003).
[57]	BMI (g = 0.67, p = 0.040) and waist to hip ratio reduced (g = 0.14, p = 0.014); bone mineral density improved (g = 0.32, p = 0.004).		
[63]	BW (g = 0.58, p<0.05), total fat mass and % fat mass decreased (g = 2.10, p<0.05).		Fasting insulin (g = 0.93, p<0.05) and ISI (g = 2.26, p<0.05) improved more in WBV. Slightly decreased leptin levels (g = 0.38, p<0.05); increase in adiponectin levels (g = 1.33, p<0.05).
[26]	BW, % body fat and lean mass of arms and legs did not change (p>0.05).	Brachial/aortic systolic (g = 0.83, p<0.001) and diastolic (g = 0.70 p = 0.008) BP decreased. Pulse pressure, augmented pressure, augmentation index (g = 0.93, p = 0.008), augmentation index adjusted to 75 bpm (g = 1.06, p = 0.002), second systolic peak and systolic tension time index decreased (g = 0.93, p<0.001).	Leg muscle strength increased (g = 0.28, p<0.001).
[54]	BW (g = 0.81, p = 0.055), visceral adipose tissue (g = 1.36, p = 0.049) and total fat mass (g = 1.17, p = 0.041) decreased.		Hand-grip, single-leg balance (g = 2.37, p<0.05) and the sit-and-reach test (g = 4.74, p<0.05) increased.
[48]		Ankle systolic BP decreased in the WBV-high group (g = 5.75, p<0.05), compared with no changes in the WBV-normal group (p>0.05). Brachial/aortic systolic BP, leg and brachial-ankle PWV similarly decreased in the WBV-high (g = 3.79, p<0.05) and WBV-normal group (g = 2.75, p<0.05).	
[59]	% Body fat decreased in both groups (g = 0.74, p). Lean mass index increased only in WBV+L (g = 1.00).	Leg and brachial-ankle PWV decreased (g = 2.75 and p<0.05 WBV, g = 2.35 and p<0.01, WBV+L). Aortic PWV decreased (g = 3.00 and p<0.01, WBV+L).	
[50]		Brachial/aortic systolic (g = 0.79 and p<0.05 WBV, g = 0.89 and p<0.01 WBV+L) and diastolic BP (g = 0.59 and p<0.05 WBV, g = 0.77 and p<0.05 WBV+L), and mean arterial pressure (g = 0.63 WBV, g = 1.00 WBV+L, p<0.05) decreased. Brachial and aortic pulse pressure decreased in WBVT+L group. AIx decreased and transit time of the reflected wave increased (g = 0.69 WBV, g = 1.10, p<0.05).	Nitric oxide concentration increased (g = 0.66 WBV, g = 0.59 WBV+L, p<0.05).
[49]	No significant changes in BW (p>0.05).	Normalized Low Frequency to normalized High Frequency ratio decreased (g = 0.75, p<0.05). Brachial systolic (g = 2.26, p<0.01) and diastolic (g = 1.70, p<0.01) BP decreased. HR, Ln of total power and of High Frequency and Low Frequency did not differ (p>0.05)	No significant changes in PASE score and dietary composition (p>0.05).
[52]	% body fat decreased (g = 1.25). No significant changes in BW (p>0.05).	HR, Ln low frequency to Ln High Frequency ratio (g = 1.80, p<0.01), and normalized Low Frequency decreased (g = 1.45, p<0.01). R-R intervals (g = 1.17, p<0.05), Ln High Frequency and normalized High Frequency increased.	No significant changes in PASE score and dietary composition; Muscle strength increased (g = 3.48, p<0.01).
[47]			Knee extension strength increased (g = 0.50, p<0.001); dynamic stability improved.
[55]		Reduction in reflexion time (g = 1.67, p<0.05). Carotid-femoral PWV reduced (g = 1.26, p<0.05). Brachial-ankle and femoral ankle PWV (g = 3.79, p<0.01), aortic systolic BP, augmented pressure and AIx adjusted to 75 bpm (g = 3.50, p<0.01) reduced.	Leg muscle strength increased (g = 5.07, p<0.001)

AIx: augmentation index; BP: blood pressure; BW: body weight; HR: heart rate; Ln: natural logarithm; PASE: Physical Activity Scale for the Elderly; PWV: pulse wave velocity; RMS: root mean square.

Twelve studies investigated the cardiovascular response to WBVT. Ten articles reported changes in arterial stiffness: systolic/diastolic BP decreased with a large effect in [26,49,55,56,60]; Figueroa et al. [48] observed a large-effect reduction in ankle systolic BP only in the hypertensive group. Carotid-femoral and/or brachial-ankle Pulse Wave Velocity (PWV) also decreased by 40–100 cm∙s^-1^ (large effects) [26,55,59,60]. Blood flow velocity increased by 35 ml∙min^-1^ with a small to medium effect [62]. The augmentation index (AIx and AIx adjusted to 75 bpm, indicators of wave reflection computed as difference between the second and first systolic peak of aortic pulse pressure) reduced (large effects) [48,49,55]. Wong et al. found that transit time of reflexion wave increased more in the group supplemented with L-Citrulline (large effect) than after WBVT alone [50]. Positive effects of WBVT in improving cardiac autonomic modulation by decreasing sympathovagal balance and/or decreasing resting heart rate (5–10 bpm) were reported consistently with large effects in young [56], healthy [52], and pre- or stage 1-hypertensive postmenopausal women [49]: low-to-high respiratory frequency power ratio (or combination thereof) improved in [49,52,56]; total power did not significantly change in [49,52] but increased in [56]; R-R intervals decreased with a large effect [52].

Three studies found hormones and hematic lipids concentrations changes following the intervention: Bellia et al. reported a decrease of fasting insulin level (-30 pmol∙l^-1^, large effect), a slight decrease in leptin levels (medium effect) and an increase in adiponectin levels (large effect) [63]. Miyaki et al. described a reduction in LDL cholesterol and triglycerides concentration (large effect) [60]. Nitric oxide (NOx) concentration increased with medium effects in [49].

Leg muscle strength improved following 6 to 12 weeks of WBVT in untrained pre- and post- menopausal obese women by 8% to 18% (large effects) [47,52,53,55], and even up to 40% [52]; So et al. revealed a large-effect improvement in hand grip (2.1±3.0 kg), single-leg balance (11.0±15.4 s) and sit-and-reach (6.5±4.8 cm) in the group undergoing WBVT and diet [54]; Yang et al. found an enhanced dynamic stability in terms of a larger decline in fall rate in WBVT (-45%) than in the placebo group (-25%) [47]. Miyaki et al. reported an increase in peak oxygen uptake (large effect); Wilms et al. found a positive effect of WBVT in enhancing resting energy expenditure (large effect) [51].

## Discussion

Six to twelve weeks of WBVT in obese individuals generally led to a reduction in fat mass and cardiovascular improvements. However, the results of the examined studies are various and sometimes inconsistent or inconclusive. Thus, they should be interpreted the light of the specific cohorts and intervention.

### Outcomes of vibration training

Sinusoidal vibrations stimulate the primary endings of the muscle spindles, which in turn activate *α*-motor neurons and induce rapid eccentric-concentric involuntary contractions; this mechanism is known as tonic vibration reflex [64]. The hypotheses about the adaptive muscular mechanisms include synchronization, stimulation of Golgi tendon organs, activation of antagonists, variation of neurotransmitter (dopamine, serotonin) concentrations [29,32]. Clearly, the reaction to vibration is not only biomechanical as WBVT elicits the combined response of the musculoskeletal, cardiovascular, endocrine and nervous systems [39].

#### Body composition

When WBVT lasted 10 weeks or more, a weight reduction was always observed [53,58,60–62]. The association between the intervention settings and the extent of weight loss is unclear: exercises performed on the plate varied between studies; side-alternating vibrations below 16 Hz produced small to moderate weight loss [57,61], as well as 40-to-60 Hz synchronous vibrations (amplitude: 2–5 mm) [53]; conversely, larger weight loss was obtained with 30-to-35 Hz vibrations [60] and after 6 weeks of WBVT at 30–35 Hz (amplitude: 2 mm) [54]. Even when body weight remained unchanged, WBVT often induced a remodelling of body composition: reduction of fat mass [58,60–63] and VAT [54,58] were observed with 8-week interventions or shorter, and with daily exposure to vibrations from 5.1 to 12.7 ms^2^ [51,52,59]. Fat mass loss was concentrated on the trunk, as proven by a reduction of VAT and waist circumferences [53,54,58].

Three factors may contribute to fat mass reduction: (i) the acute exposure to vibrations activates the central sympathetic nervous system, whose innervation of white adipose tissue triggers lipolysis [65]; (ii) WBVT enhances glycemic control by improving insulin action and glucose regulation [49,63,66]; (iii) WBVT promotes GH release [67], which stimulates metabolism and is usually reduced in obese subjects [68].

The improved glycaemic control is crucial in postmenopausal women, whose hormonal changes lead to insulin resistance [69]: Bellia et al. found a 35% increase of insulin sensitivity following 8 weeks of WBVT with static squats [63]; additional effects on metabolic regulation were an increase of adiponectin and a decrease of leptin levels [63]. In patients with type-2 Diabetes Mellitus, insulin-mediated glucose uptake in the skeletal muscle improved, probably due to increase in femoral artery blood flow [62].

Bone mineral density negatively correlates with aging and obesity [70]; after menopause, decreasing estrogen concentrations cause a further decline in bone mineral density, which can lead to osteoporosis. Fluid movement produced by vibrations is anabolic to the bone [30], as generates shear stresses on the plasma membranes of highly sensitive cells like resident osteocytes, bone lining cells and osteoblasts [71].

#### Cardiovascular effects

Eleven papers examined the role of WBVT in mitigating the adverse cardiovascular outcomes involved with obesity (i.e. increased sympathovagal balance and arterial stiffness), and complicated by aging, menopause and Diabetes. There is compelling evidence that at least 6-weeks of WBVT can reduce sympathovagal balance [49,52,56] and central/peripheral arterial stiffness [26,48,50,55,59,60] in obese women–only one paper addressed these topics in men [62].

An increase of Low Frequency (LF) and a decrease of High Frequency (HF) power of heart rate variability spectrum indicate a concurrent decrease in sympathetic and an improvement of cardiovagal modulation, respectively. This has important clinical implications since a lower sympathovagal balance (measured as LF/HF ratio or R-R duration) is associated with reduced cardiovascular risk and greater longevity in obese women [72,73]. In contrast, conventional exercise such as resistance [74] or aerobic [75] training did not improve LF or LF/HF ratio in postmenopausal women.

WBVT decreased systemic, aortic and leg arterial stiffness in terms of brachial-ankle, carotid-femoral, and femoral-ankle PWV, respectively [50,55,56,59,60]. Enhanced benefits on aortic stiffness (aortic systolic BP reduced by 8–10 mmHg) were obtained after 6 weeks of WBVT and L-citrulline supplementation in hypertensive postmenopausal women [59]. L-Citrulline is a non-protein amino acid naturally found in watermelon which is efficiently converted to L-arginine, the substrate for endothelial production of NOx [76]. Decrement in aortic systolic and diastolic BP by 5–10 mmHg corresponds to 30–40% reduction in the risk of death due to stroke and other cardiovascular complications [77]. Importantly, these hemodynamic effects of WBVT were not observed in studies following traditional resistance training in overweight and obese women [78–80]. Only high-intensity aerobic training, but not high-intensity resistance training, was effective in reducing AIx [81].

The underlying mechanisms of these outcomes rely on the combination of several factors. First, WBVT increases the levels of circulating angiotensin-II (inhibitor of cardiovagal activity [82]) and the local production of vasodilatory substances, including NOx [83]. NOx decreases systolic BP and AIx reducing the vascular tone of small arteries [84]. Second, the mechanical oscillatory contractions during vibration serve as an active muscle pump and increase stroke volume, probably enhancing venous return and preload [85]. Third, total peripheral resistance to blood flow increases during body vibration. As a compensation, more capillaries are opened to keep a necessary level of cardiac output, resulting in more efficient gas and material metabolism between the blood and muscle fibers [39]. Fourth, friction forces applied by the mechanical vibration on the endothelial cells [38] also contribute in enhancing blood flow [30,38,83]. The weekly repetition of such acute vascular effects is likely responsible for the improvements in arterial stiffness and wave reflection [56].

#### Functional and other effects

As individuals with obesity fatigue at a greater rate [86], daily motor performance may be hindered. Since increased muscle strength per se may offers protection from obesity [59], a safe, time-efficient and low-intensity exercise modality in the management of obese individuals could prevent vascular complications, muscle dysfunction and physical disability [55]. Although the training protocols differed between studies, from young obese women to the elderly WBVT produced a similar positive effect to resistance training on muscle strength [47,52,53,55,58], and an improvement in sit-and-reach and sit-to-stand functional tests [54].

Improved leg muscle blood flow can contribute to muscle mass increase in older adults [87]. With short WBVT interventions (6 weeks), Figueroa et al. [56] proposed that muscle strength gains are primarily attributed to neural adaptations rather than muscle hypertrophy. Consistently, vibration training increases the efficiency of agonist/antagonist pairs and the synchronization of motor units, which implies that more fibers are contracted at once and more force can be produced [29]. This positively affects balance control: previous observations in populations prone to fall, such as frail people [30,88,89], were confirmed in obese subjects by single leg balance improvements [54] and by a decrease in fall rate [47].

Lastly, the addition of vibrations to both static and dynamic exercises seemed to significantly increase oxygen uptake in obese women [58]. Although WBVT might increase oxygen consumption and caloric output when added to an exercise program [30,90], the amount of energy turnover due to vibration is modest, estimated as 4.5 ml·min^-1^·kg^-1^ (frequency: 26 Hz, amplitude: 3 mm) [30,91]. We argue that the potential mechanisms of increased oxygen uptake could be the higher metabolism due to hormonal and cardiovascular changes [39,67], increase of lean mass and muscle activation [91].

### Side effects

The effects of vibration training on human body may depend on vibration settings (frequency, amplitude and duration) and on the exercise program (type of exercises, intensity and volume). WBVT stimulates reflexive muscle contractions “in a safe and gentle manner” [52] depending on the vibration settings: vibrations ranged from tolerable levels (6 papers, typically 1–2 mm at 25–40 Hz, 5.1 m·s^-2^), to several times higher than what the ISO 2631–1:1997 considers a safe threshold for workers (8 papers, up to 42 m·s^-2^). It is worth noting that ISO 2631–1:1997 thresholds were established to minimize health threats deriving from a continuous vibration exposure in the workplace, and may not be directly transferable to medical devices and WBVT [30,92]. Six papers explicitly reported no unfavourable symptoms or adverse effects resulting from the vibration stimulus [47,50,54,55,61,93]. However, isolated cases of lower leg phlebitis [58], mild knee pain [94] and back pain after two weeks of training [63] were reported.

In summary, while it is presumed that the benefits of using vibrating devices offset the risks generated by exposure [92], the use of WBVT for therapeutic purposes is still not standardized and the related potential adverse effects are uncertain [51], especially the implications on brain health due to chronic exposure to accelerations. Some guidelines can be drawn, though. First, Muir et al. proposed that vibrations delivered by medical devices can be considered reasonably safe on a basis of 15 minutes of exposure/day if enclosed within the boundaries of 30–50 Hz and 2.25–7.98 g [92]; consistently, in all the reviewed papers the daily duration of training was lower than 10–15 min. Second, vibrations close to the main resonant natural frequency of the human body (5–20 Hz) can produce accelerations to the head equalling or exceeding the g-force delivered at the foot, and should be prudently avoided [39]. Third, transmissibility to the cranium is markedly attenuated by flexing the knees and by using side-alternating rather than synchronous vibration [92]; thus, straight-legged stance has to be avoided.

### Limitations

Five major flaws emerged in the assessed papers: (i) few comparison studies, (ii) small sample sizes, (iii) potential sex and regional biases, (iv) poor quality of WBVT reporting and (v) lack of long-term investigations.

- While evidence of WBVT effectiveness exists when compared to not-exercising control groups [26,48,49,53,55,61], relevant therapeutic information could come from the comparison of different WBVT settings and exercise modalities. Unfortunately, only three studies compared WBVT to other training [51,54,63], and no study addressed the effect of different vibration settings on obese individuals.
- Conventionally, the number of independent variables (k) can be considered adequate if the ratio k:n exceeds 1:10, where n is the sample size [95]. No article included in this review met such requirement, as all papers but [57] involved less than 26 obese subjects. Small sample sizes reduce the relevance of the results by increasing the risk of type-II error and reducing the ability to validate hypotheses. Further, small sample size did not allow to evaluate different intervention settings and exercises, as admitted by [49,63,94].
- Hemodynamic effects [96], systolic BP and AIx [13,15], insulin and GH hormones concentration present gender-specific differences. This limits the generalization of results found on women, as WBVT may not have the same effects on obese men [93]. Further, some results were attributed to very specific cohorts (i.e. Korean [94] or Hispanic [55]), and cannot be generalized to any obese population due to regional-specific features of minority racial/ethnic groups, as the higher incidence of reduced HR variability in Hispanics [97].
- Given the potential damage of WBVT training, supervised use of devices by trained therapists should be recommended [92]. It is likewise essential to describe whether study participants were standing freely on the device or were holding on to some support, and the type of footwear, which influences the transmissibility to the body structures [43]. However, a third of the examined paper did not mention any supervision; type of support was indicated in five studies and footwear in two; skidding and accuracy of vibration parameters was never reported. These details may have introduced additional variability and played a role as confounding factors.
- Lastly, 11/18 papers presented results after 6 or 8 weeks, a relatively short period of intervention; how long beneficial effects persist after the intervention remains an open question, like the potential risk of WBVT-related side effects in the long term [63]. Results by Vissers et al. [58], showing that patients treated with WBVT succeeded in maintaining a weight loss of 10% at 12 months, are encouraging but need to be more extensively confirmed.

## Conclusions and recommendations for future research

Whole-body vibration training is a promising adjuvant intervention therapy for obese women. In particular, there is evidence that at least 6 weeks of WBVT can improve cardiac autonomic function [49,52] and reduce central and peripheral arterial stiffness [50,55]; 10 or more weeks of WBVT produces significant body weight drop [53,58,60–62], leg strength improvements [48,52,53,55], and in addition to hypocaloric diet may further enhance insulin-sensitivity and glucose regulation [63].

WBVT could be prescribed without additional exercise in the initial stages of a weight loss program, due to the limited stress upon joints and the GH-mediated stimulation of metabolism, without provoking an excessive fatigue [63,85]. Passive vibrations do not involve voluntary movement and require a lower contribution of central command [98]. Thus, WBVT could be a useful mode of exercise for deconditioned obese with poor motivation [56]: when combined with dietary intervention or prescribed as alternative to traditional exercise training, WBVT is as effective as aerobic and resistance exercise in reducing fat mass [58,93] and moderating the deficit of the relative muscle strength [49,54]. Lastly, WBVT may be effective in vascular health promotion and prevention in young obese women [50,55,59].

The positive potential of WBVT for obese individuals is partly hindered by methodological inconsistencies in the existing literature. The lack of results on obese males, and the small (and occasionally biased) cohorts suggest further research to standardise WBVT in the management of obesity. Two additional main directions of research emerged: first, it is mandatory to make light on the causality between the vibration parameters (frequency, amplitude, exposure, work:rest ratio) and the WBVT outcomes. Second, long-term studies are required to demonstrate safety and health benefit that can be achieved with WBVT for obese patients.

## Supporting information

S1 Table Methodological quality of the examined randomized controlled trials according to the PEDro scale (Y: yes, N: no or not applicable). (DOCX)

S2 Table Methodological quality of the examined non-randomized studies according to the TREND scale (Y: yes, N: no or not applicable). (DOCX)

S3 Table Quality of reporting whole-body vibration treatment (Y: yes, N: no or not applicable). (DOCX)

S4 Table Preferred reporting guidelines for systematic reviews and meta-analysis (PRISMA) checklist [40]. (PDF)

## References

[1] Hales CM, Fryar CD, Carroll MD, Freedman DS, Ogden CL. Trends in Obesity and Severe Obesity Prevalence in US Youth and Adults by Sex and Age, 2007–2008 to 2015–2016. JAMA. 2018; 10.1001/jama.2018.3060 29570750 PMC5876828

[2] Adams KF, Schatzkin A, Harris TB, Kipnis V, Mouw T, Ballard-Barbash R, et al Overweight, obesity, and mortality in a large prospective cohort of persons 50 to 71 years old. N Engl J Med. 2006;355: 763–78. 10.1056/NEJMoa055643 16926275

[3] Yumuk V, Tsigos C, Fried M, Schindler K, Busetto L, Micic D, et al European Guidelines for Obesity Management in Adults. Obes Facts. 2015;8: 402–424. 10.1159/000442721 26641646 PMC5644856

[4] Bogers R, Bemelmans W, Hoogenveen R, Boshuizen H, Woodward M, Knekt P, et al Association of overweight with increased risk of coronary heart disease partly independent of blood pressure and cholesterol levels. Arch Intern Med. 2007;167: 1720–1728. 10.1001/archinte.167.16.1720 17846390

[5] Croymans DM, Krell SL, Oh CS, Katiraie M, Lam CY, Harris RA, et al Effects of resistance training on central blood pressure in obese young men. J Hum Hypertens. 2014;28: 157–164. 10.1038/jhh.2013.81 24005959 PMC4119468

[6] Koenig J, Jarczok MN, Warth M, Ellis RJ, Bach C, Hillecke TK, et al Body mass index is related to autonomic nervous system activity as measured by heart rate variability—a replication using short term measurements. J Nutr Health Aging. 2014;18: 300–2. 10.1007/s12603-014-0022-6 24626758 PMC12880227

[7] Baek J, Park D, Kim I, Won JU, Hwang J, Roh J. Autonomic dysfunction of overweight combined with low muscle mass. Clin Auton Res. 2013;23: 325–331. 10.1007/s10286-013-0215-9 24221882

[8] McGee DL. Body mass index and mortality: A meta-analysis based on person-level data from twenty-six observational studies. Ann Epidemiol. 2005;15: 87–97. 10.1016/j.annepidem.2004.05.012 15652713

[9] Strazzullo P, D’Elia L, Cairella G, Garbagnati F, Cappuccio FP, Scalfi L. Excess body weight and incidence of stroke: Meta-analysis of prospective studies with 2 million participants. Stroke. 2010 10.1161/STROKEAHA.109.576967 20299666

[10] Himes CL, Reynolds SL. Effect of obesity on falls, injury, and disability. J Am Geriatr Soc. 2012;60: 124–129. 10.1111/j.1532-5415.2011.03767.x 22150343

[11] Fjeldstad C, Fjeldstad AS, Acree LS, Nickel KJ, Gardner AW. The influence of obesity on falls and quality of life. Dyn Med. 2008;7 10.1186/1476-5918-7-4 18304350 PMC2288598

[12] Wu X, Lockhart TE, Yeoh HT. Effects of obesity on slip-induced fall risks among young male adults. J Biomech. 2012;45: 1042–1047. 10.1016/j.jbiomech.2011.12.021 22304846 PMC3310324

[13] Russo C, Jin Z, Palmieri V, Homma S, Rundek T, Elkind MSV, et al Arterial stiffness and wave reflection: Sex differences and relationship with left ventricular diastolic function. Hypertension. 2012;60: 362–368. 10.1161/HYPERTENSIONAHA.112.191148 22753223 PMC3402954

[14] Wildman RP, Mackey RH, Bostom A, Thompson T, Sutton-Tyrrell K. Measures of obesity are associated with vascular stiffness in young and older adults. Hypertension. 2003;42: 468–473. 10.1161/01.HYP.0000090360.78539.CD 12953016

[15] Shim CY, Park S, Choi D, Yang W-I, Cho I-J, Choi E-Y, et al Sex differences in central hemodynamics and their relationship to left ventricular diastolic function. J Am Coll Cardiol. 2011;57: 1226–1233. 10.1016/j.jacc.2010.09.067 21371640

[16] Palmer BF, Clegg DJ. The sexual dimorphism of obesity. Molecular and Cellular Endocrinology. 2015 pp. 113–119. 10.1016/j.mce.2014.11.029 25578600 PMC4326001

[17] Wadden TA, Butryn ML, Byrne KJ. Efficacy of Lifestyle Modification for Long-Term Weight Control. Obes Res. 2004;12: 151S–162S. 10.1038/oby.2004.282 15687411

[18] Matarese LE, Pories WJ. Adult weight loss diets: Metabolic effects and outcomes. Nutrition in Clinical Practice. 2014 pp. 759–767. 10.1177/0884533614550251 25293593

[19] Irving BA, Davis CK, Brock DW, Weltman JY, Swift D, Barrett EJ, et al Effect of exercise training intensity on abdominal visceral fat and body composition. Med Sci Sports Exerc. 2008;40: 1863–1872. 10.1249/MSS.0b013e3181801d40 18845966 PMC2730190

[20] Kelley CP, Sbrocco G, Sbrocco T. Behavioral Modification for the Management of Obesity. Primary Care—Clinics in Office Practice. 2016 pp. 159–175. 10.1016/j.pop.2015.10.004 26896208 PMC4772897

[21] Andrade AM, Coutinho SR, Silva MN, Mata J, Vieira PN, Minderico CS, et al The effect of physical activity on weight loss is mediated by eating self-regulation. Patient Educ Couns. 2010;79: 320–326. 10.1016/j.pec.2010.01.006 20149955

[22] Janssen I, Ross R. Effects of sex on the change in visceral, subcutaneous adipose tissue and skeletal muscle in response to weight loss. Int J Obes Relat Metab Disord. 1999;23: 1035–1046. 10557024 10.1038/sj.ijo.0801038

[23] Gerage AM, Forjaz CLM, Nascimento MA, Januário RSB, Polito MD, Cyrino ES. Cardiovascular adaptations to resistance training in elderly postmenopausal women. Int J Sports Med. 2013;34: 806–813. 10.1055/s-0032-1331185 23459854

[24] Willis LH, Slentz CA, Bateman LA, Shields AT, Piner LW, Bales CW, et al Effects of aerobic and/or resistance training on body mass and fat mass in overweight or obese adults. J Appl Physiol. 2012;113: 1831–1837. 10.1152/japplphysiol.01370.2011 23019316 PMC3544497

[25] Guérin E, Fortier MS. Situational motivation and perceived intensity: Their interaction in predicting changes in positive affect from physical activity. J Obes. 2012;2012. 10.1155/2012/269320 22778914 PMC3388456

[26] Figueroa A, Kalfon R, Wong A. Whole-body vibration training decreases ankle systolic blood pressure and leg arterial stiffness in obese postmenopausal women with high blood pressure. Menopause. 2014;22: 1–5. 10.1097/GME.0000000000000332 25225715

[27] Machado A, García-López D, González-Gallego J, Garatachea N. Whole-body vibration training increases muscle strength and mass in older women: A randomized-controlled trial. Scand J Med Sci Sports. 2010;20: 200–207. 10.1111/j.1600-0838.2009.00919.x 19422657

[28] Roelants M, Delecluse C, Verschueren SM. Whole-body-vibration training increases knee-extension strength and speed of movement in older women. J Am Geriatr Soc. 2004;52: 901–908. 10.1111/j.1532-5415.2004.52256.x 15161453

[29] Cardinale M, Bosco C. The use of vibration as an exercise intervention. Exercise and Sport Sciences Reviews. 2003 pp. 3–7. 10.1097/00003677-200301000-00002 12562163

[30] Rittweger J. Vibration as an exercise modality: How it may work, and what its potential might be. European Journal of Applied Physiology. 2010 pp. 877–904. 10.1007/s00421-009-1303-3 20012646

[31] Cochrane DJ. Vibration exercise: The potential benefits. Int J Sports Med. 2011;32: 75–99. 10.1055/s-0030-1268010 21165804

[32] Delecluse C, Roelants M, Verschueren S. Strength increase after whole-body vibration compared with resistance training. Med Sci Sports Exerc. 2003;35: 1033–1041. 10.1249/01.MSS.0000069752.96438.B0 12783053

[33] Rubin CT, Capilla E, Luu YK, Busa B, Crawford H, Nolan DJ, et al Adipogenesis is inhibited by brief, daily exposure to high-frequency, extremely low-magnitude mechanical signals. Proc Natl Acad Sci. 2007;104: 17879–17884. 10.1073/pnas.0708467104 17959771 PMC2077057

[34] Maddalozzo GF, Iwaniec UT, Turner RT, Rosen CJ, Widrick JJ. Whole-body vibration slows the acquisition of fat in mature female rats. Int J Obes. 2008;32: 1348–1354. 10.1038/ijo.2008.111 18663370 PMC2586051

[35] Totosy de Zepetnek JO, Giangregorio LM, Craven BC. Whole-body vibration as potential intervention for people with low bone mineral density and osteoporosis: a review. J Rehabil Res Dev. 2009;46: 529–542. 10.1682/JRRD.2008.09.0136 19882487

[36] Prisby RD, Lafage-Proust MH, Malaval L, Belli A, Vico L. Effects of whole body vibration on the skeleton and other organ systems in man and animal models: What we know and what we need to know. Ageing Res Rev. 2008;7: 319–329. 10.1016/j.arr.2008.07.004 18762281

[37] Bogaerts ACG, Delecluse C, Claessens AL, Troosters T, Boonen S, Verschueren SMP. Effects of whole body vibration training on cardiorespiratory fitness and muscle strength in older individuals (a 1-year randomised controlled trial). Age Ageing. 2009;38: 448–454. 10.1093/ageing/afp067 19439517

[38] Lohman EB, Petrofsky JS, Maloney-Hinds C, Betts-Schwab H, Thorpe D. The effect of whole body vibration on lower extremity skin blood flow in normal subjects. Med Sci Monit. 2007;13: CR71–6. 17261985

[39] Mester J, Kleinöder H, Yue Z. Vibration training: Benefits and risks. J Biomech. 2006;39: 1056–1065. 10.1016/j.jbiomech.2005.02.015 15869759

[40] Moher D, Shamseer L, Clarke M, Ghersi D, Liberati A, Petticrew M, et al Preferred reporting items for systematic review and meta-analysis protocols (PRISMA-P) 2015 statement. Syst Rev. 2015;4 10.1186/2046-4053-4-1 25554246 PMC4320440

[41] Fitzpatrick RB. PEDro: A physiotherapy evidence database. Med Ref Serv Q. 2008;27: 188–197. 10.1080/02763860802114397 18844091

[42] Des Jarlais DC, Lyles C, Crepaz N. Improving the Reporting Quality of Nonrandomized Evaluations of Behavioral and Public Health Interventions: The TREND Statement. American Journal of Public Health. 2004 10.2105/AJPH.94.3.361 PMC1448256 14998794

[43] Rauch F, Sievanen H, Boonen S, Cardinale M, Degens H, Felsenberg D, et al Reporting whole-body vibration intervention studies: Recommendations of the International Society of Musculoskeletal and Neuronal Interactions. J Musculoskelet Neuronal Interact. 2010;10: 193–198. 20811143

[44] Pietro Emerenziani G, Meucci M, Gallotta MC, Buzzachera CF, Guidetti L, Baldari C. Whole body vibration: unsupervised training or combined with a supervised multi-purpose exercise for fitness? J Sports Sci. 2014;32: 1033–1041. 10.1080/02640414.2013.877150 24479642

[45] International Standards Organization. ISO 2631–1: Mechanical Vibration and shock- Evaluation of human exposure to whole-body vibration- Part 1- General Requirements. Order A Journal On The Theory Of Ordered Sets And Its Applications 1997.

[46] Durlak JA. How to select, calculate, and interpret effect sizes. Journal of Pediatric Psychology. 2009 pp. 917–928. 10.1093/jpepsy/jsp004 19223279

[47] Yang F, Munoz J, zhu Han L, Yang F. Effects of vibration training in reducing risk of slip-related falls among young adults with obesity. J Biomech. 2017;57: 87–93. 10.1016/j.jbiomech.2017.03.024 28431747

[48] Figueroa A, Kalfon R, Madzima TA, Wong A. Effects of whole-body vibration exercise training on aortic wave reflection and muscle strength in postmenopausal women with prehypertension and hypertension. J Hum Hypertens. 2014;28: 118–122. 10.1038/jhh.2013.59 23823582

[49] Wong A, Alvarez-Alvarado S, Kinsey AW, Figueroa A. Whole-Body Vibration Exercise Therapy Improves Cardiac Autonomic Function and Blood Pressure in Obese Pre- and Stage 1 Hypertensive Postmenopausal Women. J Altern Complement Med. 2016;22: 970–976. 10.1089/acm.2016.0124 27656953

[50] Wong A, Alvarez-Alvarado S, Jaime SJ, Kinsey AW, Spicer MT, Madzima TA, et al Combined whole-body vibration training and l-citrulline supplementation improves pressure wave reflection in obese postmenopausal women. Appl Physiol Nutr Metab = Physiol Appl Nutr métabolisme. 2016;41: 292–7. 10.1139/apnm-2015-0465 26863234

[51] Wilms B, Frick J, Ernst B, Mueller R, Wirth B, Schultes B. Whole body vibration added to endurance training in obese women—A pilot study. Int J Sports Med. 2012;33: 740–743. 10.1055/s-0032-1306284 22562734

[52] Severino G, Sanchez-Gonzalez M, Walters-Edwards M, Nordvall M, Chernykh O, Adames J, et al Whole-body vibration training improves heart rate variability and body fat percentage in obese hispanic postmenopausal women. J Aging Phys Act. 2017;25: 395–401. 10.1123/japa.2016-0087 27918705

[53] Milanese C, Piscitelli F, Zenti MG, Moghetti P, Sandri M, Zancanaro C. Ten-week whole-body vibration training improves body composition and muscle strength in obese women. Int J Med Sci. 2013;10: 307–311. 10.7150/ijms.5161 23423629 PMC3575626

[54] So R, Eto M, Tsujimoto T, Tanaka K. Acceleration training for improving physical fitness and weight loss in obese women. Obes Res Clin Pract. 2014;8: e201–e98. 10.1016/j.orcp.2013.03.002 24847665

[55] Alvarez-Alvarado S, Jaime SJ, Ormsbee MJ, Campbell JC, Post J, Pacilio J, et al Benefits of whole-body vibration training on arterial function and muscle strength in young overweight/ obese women. Hypertens Res. 2017;40: 487–492. 10.1038/hr.2016.178 28077859

[56] Figueroa A, GIl R, Wong A, Hooshmand S, Park SY, Vicil F, et al Whole-body vibration training reduces arterial stiffness, blood pressure and sympathovagal balance in young overweight/obese women. Hypertens Res. 2012;35: 667–672. 10.1038/hr.2012.15 22357522

[57] Zaki ME. Effects of whole body vibration and resistance training on bone mineral density and anthropometry in obese postmenopausal women. J Osteoporos. 2014;2014. 10.1155/2014/702589 25136473 PMC4086652

[58] Vissers D, Verrijken A, Mertens I, Van Gils C, Van De Sompel A, Truijen S, et al Effect of long-term whole body vibration training on visceral adipose tissue: A preliminary report. Obes Facts. 2010;3: 93–100. 10.1159/000301785 20484941 PMC6452127

[59] Figueroa A, Alvarez-Alvarado S, Ormsbee MJ, Madzima TA, Campbell JC, Wong A. Impact of l-citrulline supplementation and whole-body vibration training on arterial stiffness and leg muscle function in obese postmenopausal women with high blood pressure. Exp Gerontol. 2015;63: 35–40. 10.1016/j.exger.2015.01.046 25636814

[60] Miyaki A, Maeda S, Choi Y, Akazawa N, Tanabe Y, So R, et al The addition of whole-body vibration to a lifestyle modification on arterial stiffness in overweight and obese women. Artery Res. 2012;6: 85–91. 10.1016/j.artres.2012.01.006

[61] Adsuar J, B Del Pozo-Cruz, Parraca J, Corzo H, Olivares P, Gusi N. Vibratory Exercise Training Effects on Weight in Sedentary Women with Fibromyalgia. Int J Med Sci Phys Act Sport. 2013;13: 295–305.

[62] Sañudo B, Alfonso-Rosa R, Del Pozo-Cruz B, Del Pozo-Cruz J, Galiano D, Figueroa A. Whole body vibration training improves leg blood flow and adiposity in patients with type 2 diabetes mellitus. Eur J Appl Physiol. 2013;113: 2245–2252. 10.1007/s00421-013-2654-3 23657766

[63] Bellia A, Sallì M, Lombardo M, D’Adamo M, Guglielmi V, Tirabasso C, et al Effects of whole body vibration plus diet on insulin-resistance in middle-aged obese subjects. Int J Sports Med. 2014;35: 511–516. 10.1055/s-0033-1354358 24227120

[64] Kvorning T, Bagger M, Caserotti P, Madsen K. Effects of vibration and resistance training on neuromuscular and hormonal measures. Eur J Appl Physiol. 2006;96: 615–625. 10.1007/s00421-006-0139-3 16482475

[65] Snitker S, Macdonald I, Ravussin E, Astrup A. The sympathetic nervous system and obesity: role in aetiology and treatment. Obes Rev. 2000;1: 5–15. 12119646 10.1046/j.1467-789x.2000.00001.x

[66] Di Loreto C, Ranchelli A, Lucidi P, Murdolo G, Parlanti N, De Cicco A, et al Effects of whole-body vibration exercise on the endocrine system of healthy men. J Endocrinol Invest. 2004;27: 323–327. 10.1007/BF03351056 15233550

[67] Giunta M, Cardinale M, Agosti F, Patrizi A, Compri E, Rigamonti AE, et al Growth hormone-releasing effects of whole body vibration alone or combined with squatting plus external load in severely obese female subjects. Obes Facts. 2012;5: 567–574. 10.1159/000342066 22922806

[68] Sartorio A, Spada A, Morabito F, Faglia G. Different GH responsiveness to repeated GHRH administration in normal children and adults. J Endocrinol Invest. 1988;11: 727–729. 10.1007/BF03350930 3148001

[69] Piché ME, Lapointe A, Weisnagel SJ, Corneau L, Nadeau A, Bergeron J, et al Regional body fat distribution and metabolic profile in postmenopausal women. Metabolism. 2008;57: 1101–1107. 10.1016/j.metabol.2008.03.015 18640388

[70] Premaor MO, Enrsud K, Lui L, Parker RA, Cauley J, Hillier TA, et al Risk factors for nonvertebral fracture in obese older women. J Clin Endocrinol Metab. 2011;96: 2414–2421. 10.1210/jc.2011-0076 21677038 PMC3146794

[71] Rubin C, Turner AA, Mallinckrodt C, Jerome C, McLeod K, Bain S. Mechanical strain, induced noninvasively in the high-frequency domain, is anabolic to cancellous bone, but not cortical bone. Bone. 2002;30: 445–452. 11882457 10.1016/s8756-3282(01)00689-5

[72] Pal G, Chandrasekaran A, Haribaran A. Body mass index contributes to sympathovagal imbalance in pre- hypertensive. BMC Cardiovasc Disord. 2012;12: 54 10.1186/1471-2261-12-54 22812583 PMC3441642

[73] Tadic C, Cuspidi C, Pencic B, Marjanovic T, Celic V. The association between heart rate variability and biatrial phasic function in arterial hypertension. J Am Soc Hypertens. 2014;8: 699–708. 10.1016/j.jash.2014.07.032 25418491

[74] Ryan AS, Pratley RE, Elahi D, Goldberg AP. Resistive training increases fat-free mass and maintains RMR despite weight loss in postmenopausal women. J Appl Physiol. 1995;79: 818–823. 10.1152/jappl.1995.79.3.818 8567523

[75] Jurca R, Church TS, Morss GM, Jordan AN, Earnest CP. Eight weeks of moderate-intensity exercise training increases heart rate variability in sedentary postmenopausal women. Am Heart J. 2004;147: e8–e15. 10.1016/j.ahj.2003.09.015 15131556

[76] Schwedhelm E, Maas R, Freese R, Jung D, Lukacs Z, Jambrecina A, et al Pharmacokinetic and pharmacodynamic properties of oral L-citrulline and L-arginine: Impact on nitric oxide metabolism. Br J Clin Pharmacol. 2008;65: 51–59. 10.1111/j.1365-2125.2007.02990.x 17662090 PMC2291275

[77] Vlachopoulos C, Aznaouridis K, O’Rourke MF, Safar ME, Baou K, Stefanadis C. Prediction of cardiovascular events and all-cause mortality with central haemodynamics: A systematic review and meta-analysis. Eur Heart J. 2010;31: 1865–1871. 10.1093/eurheartj/ehq024 20197424

[78] Beck DT, Martin JS, Casey DP, Braith RW. Exercise training reduces peripheral arterial stiffness and myocardial oxygen demand in young prehypertensive subjects. Am J Hypertens. 2013;26: 1093–1102. 10.1093/ajh/hpt080 23736111 PMC3741227

[79] Rossow LM, Fahs CA, Thiebaud RS, Loenneke JP, Kim D, Mouser JG, et al Arterial stiffness and blood flow adaptations following eight weeks of resistance exercise training in young and older women. Exp Gerontol. 2014;53: 48–56. 10.1016/j.exger.2014.02.010 24566193

[80] Figueroa A, Vicili F, Sanchez-Gonzalez M, Wong A, Ormsbee MJ, Hooshmand S. Effects of diet and/or low-intensity resistance exercise training on arterial stiffness, adiposity. J Hypertens. 2013;26: 416–423. 10.1093/ajh/hps050 23382493

[81] Ashor AW, Lara J, Siervo M, Celis-Morales C, Mathers JC. Effects of exercise modalities on arterial stiffness and wave reflection: a systematic review and meta-analysis of randomized controlled trials. PLoS One. 2014;9: e110034 10.1371/journal.pone.0110034 25333969 PMC4198209

[82] Townend JN, Al-Ani M, West JN, Littler WA, Coote JH. Modulation of cardiac autonomic control in humans by angiotensin II. Hypertension. 1995;25: 1270–1275. 7768573 10.1161/01.hyp.25.6.1270

[83] Maloney-Hinds C, Petrofsky JS, Zimmerman G, Hessinger DA. The role of nitric oxide in skin blood flow increases due to vibration in healthy adults and adults with type 2 diabetes. Diabetes Tecnol Ther. 2009;11: 39–43. 10.1089/dia.2008.0011 19132854

[84] Kelly RP, Millasseau SC, Ritter JM, Chowienczyk PJ. Vasoactive drugs influence aortic augmentation index independently of pulse-wave velocity in healthy men. Hypertension. 2001;37: 1429–1433. 11408390 10.1161/01.hyp.37.6.1429

[85] Dipla K, Kousoula D, Zafeiridis A, Karatrantou K, Nikolaidis MG, Kyparos A, et al Exaggerated haemodynamic and neural responses to involuntary contractions induced by whole-body vibration in normotensive obese versus lean women. Exp Physiol. 2016;101: 717–730. 10.1113/EP085556 27061448

[86] Maffiuletti NA, Jubeau M, Munzinger U, Bizzini M, Agosti F, De Col A, et al Differences in quadriceps muscle strength and fatigue between lean and obese subjects. Eur J Appl Physiol. 2007;101: 51–59. 10.1007/s00421-007-0471-2 17476522

[87] Dillon EL, Casperson SL, Durham WJ, Randolph KM, Urban RJ, Volpi E, et al Muscle protein metabolism responds similarly to exogenous amino acids in healthy younger and older adults during NO-induced hyperemia. AJP Regul Integr Comp Physiol. 2011;301: R1408–R1417. 10.1152/ajpregu.00211.2011 21880862 PMC3213950

[88] Yang F, King GA, Dillon L, Su X. Controlled whole-body vibration training reduces risk of falls among community-dwelling older adults. J Biomech. 2015;48: 3206–3212. 10.1016/j.jbiomech.2015.06.029 26189095

[89] Sañudo B, Carrasco L, Hoyo M, Oliva-Pascual-Vaca Á, Rodríguez-Blanco C. Changes in body balance and functional performance following whole-body vibration training in patients with fibromyalgia syndrome: A randomized controlled trial. J Rehabil Med. 2013;45: 678–684. 10.2340/16501977-1174 23828124

[90] Signorile J. Whole body vibration, part one: what’s shakin’ now? J Act Aging. 2011;10: 46–59.

[91] Vissers D, Baeyens J-P, Truijen S, Ides K, Vercruysse C-C, Van Gaal L. The Effect of Whole Body Vibration Short-Term Exercises on Respiratory Gas Exchange in Overweight and Obese Women. Phys Sportsmed. 2009;37: 88–94. 10.3810/psm.2009.10.1733 20048532

[92] J M, Kiel DP, Rubin CT. Safety and severity of accelerations delivered from whole body vibration exercise devices to standing adults. J Sci Med Sport. 2013;16: 526–531. 10.1016/j.jsams.2013.01.004 23453990 PMC3688642

[93] Nam S-S, Sunoo S, Park H-Y, Moon H-W. The effects of long-term whole-body vibration and aerobic exercise on body composition and bone mineral density in obese middle-aged women. J Exerc Nutr Biochem. 2016;20: 19–27. doi: 10.20463/jenb.2016.06.20.2.3 27508150 10.20463/jenb.2016.06.20.2.3 PMC4977903

[94] Song G-E, Kim K, Lee D-J, Joo N-S. Whole Body Vibration Effects on Body Composition in the Postmenopausal Korean Obese Women: Pilot Study. Korean J Fam Med. 2011;32: 399 10.4082/kjfm.2011.32.7.399 22745878 PMC3383152

[95] van der Linde H, Hofstad CJ, Geurts ACH, Postema K, Geertzen JHB, van Limbeek J. A systematic literature review of the effect of different prosthetic components on human functioning with a lower-limb prosthesis. J Rehabil Res Dev. 2004;41: 555–570. 10.1682/JRRD.2003.06.0102 15558384

[96] Ives SJ, McDaniel J, Witman MAH, Richardson RS. Passive limb movement: evidence of mechanoreflex sex specificity. AJP Hear Circ Physiol. 2013;304: H154–H161. 10.1152/ajpheart.00532.2012 23086995 PMC3543682

[97] Lampert R, Ickovics J, Horwitz R, Lee F. Depressed autonomic nervous system function in African Americans and individuals of lower social class: A potential mechanism of race- and class-related disparities in health outcomes. Am Heart J. 2005;150: 153–160. 10.1016/j.ahj.2004.08.008 16084163

[98] Ogoh S, Fisher JP, Dawson EA, White MJ, Secher NH, Raven PB. Autonomic nervous system influence on arterial baroreflex control of heart rate during exercise in humans. J Physiol. 2005;566: 599–611. 10.1113/jphysiol.2005.084541 15890708 PMC1464761

