# Supplementary material for: Whole-body vibration training in obese subjects: A systematic review
Source: PLoS One. 2018 Sep 5;13(9):e0202866. doi: 10.1371/journal.pone.0202866 (PMC6124767; doi:10.1371/journal.pone.0202866)
Supplement: S1 Table — (DOCX) [file pone.0202866.s001.docx]

S1 Table: methodological quality of the examined randomized controlled trials according to the PEDro scale (Y: yes, N: no or not applicable).

| **Reference** | **Year** | **Eligibility** | **Random allocation** | **Concealed allocation** | **Baseline comparability** | **Blind subjects** | **Blind therapists** | **Blind assessors** | **Measures for at least 85% of subjects** | **Intention to treat** | **Between group comparison** | **Point estimates and variability** | **TOT** |
| --- | --- | --- | --- | --- | --- | --- | --- | --- | --- | --- | --- | --- | --- |
| [58] | 2010 | Y | Y | N | Y | N | N | N | N | N | Y | Y | **4** |
| [94] * | 2011 | Y | N | N | N | N | N | Y | Y | N | N | N | **3** |
| [56] | 2012 | N | Y | N | Y | N | N | N | Y | N | Y | Y | **5** |
| [51] | 2012 | N | Y | N | Y | N | N | N | N | N | Y | Y | **4** |
| [61] | 2013 | Y | Y | Y | Y | N | N | N | Y | Y | Y | Y | **7** |
| [62] | 2013 | N | Y | N | Y | N | N | Y | N | N | Y | Y | **5** |
| [53] | 2013 | N | Y | N | Y | N | N | N | N | N | Y | Y | **4** |
| [57] | 2014 | Y | Y | Y | Y | N | N | N | Y | Y | Y | Y | **7** |
| [63] | 2014 | Y | Y | Y | Y | N | N | N | Y | N | Y | Y | **6** |
| [26] | 2014 | Y | Y | N | Y | N | N | Y | Y | N | Y | Y | **6** |
| [48] | 2014 | N | Y | N | Y | N | N | N | Y | N | Y | Y | **5** |
| [59] | 2015 | Y | Y | N | Y | N | N | N | Y | N | Y | Y | **6** |
| [50] | 2016 | Y | Y | N | Y | N | N | N | N | N | Y | Y | **4** |
| [49] | 2016 | Y | Y | N | Y | N | N | N | Y | Y | Y | Y | **6** |
| [52] | 2016 | Y | Y | N | Y | N | N | N | Y | N | Y | Y | **5** |
| [47] | 2017 | N | Y | N | Y | N | N | N | Y | Y | Y | Y | **6** |
| [55] | 2017 | Y | Y | N | Y | N | N | N | Y | Y | Y | Y | **6** |
|  | Total* | 12 | 16 | 4 | 17 | 0 | 0 | 2 | 13 | 8 | 18 | 18 |  |

* The excluded paper was not counted.
