# Supplementary material for: Whole-body vibration training in obese subjects: A systematic review
Source: PLoS One. 2018 Sep 5;13(9):e0202866. doi: 10.1371/journal.pone.0202866 (PMC6124767; doi:10.1371/journal.pone.0202866)
Supplement: S2 Table — (DOCX) [file pone.0202866.s002.docx]

S2 Table: methodological quality of the examined non-randomized studies according to the TREND scale (Y: yes, N: no, N/A: not applicable).

| **Paper Session / Topic** | **Item n.** | **Descriptor (abbreviation)** | **Miyaki et al.** [60] | **So et al.** [54] |
| --- | --- | --- | --- | --- |
| Title and abstract | 1 | Title: unit allocations | N | Y |
|  |  | Title: structured abstract | Y | Y |
|  |  | Information on target population | Y | Y |
| Introduction | 2 | Background and explanation | Y | Y |
|  |  | Theories used | Y | Y |
| Methods: participants | 3 | Methods: eligibility | Y | Y |
|  |  | Method of recruitment | N | Y |
|  |  | Recruitment setting | N | Y |
|  |  | Settings and location of data collection | N | N |
| Methods: interventions | 4 | Details | Y | Y |
|  |  | Content | Y | Y |
|  |  | Delivery method | Y | Y |
|  |  | Deliverer | N | Y |
|  |  | Setting | N | N |
|  |  | Exposure and duration | Y | Y |
|  |  | Time span | Y | Y |
|  |  | Activities to increase compliance | N | N |
| Methods: objectives | 5 | Specific objectives and hypothesis | Y | Y |
| Methods: outcomes | 6 | Clearly defined outcomes | N | N |
|  |  | Methods used to collect data | Y | Y |
|  |  | Information on validated instruments | N | N |
| Methods: sample size | 7 | Sample size determination | N | N |
| Methods: assignment | 8 | Unit of assignment | Y | Y |
|  |  | Method of assignment | Y | Y |
|  |  | Aspects employed to minimize potential bias | N | N |
| Methods: blinding | 9 | Masking | N | N |
| Methods: unit of analysis | 10 | Unit description | Y | Y |
|  |  | Unit adjustment | N | N |
| Methods: statistical methods | 11 | Statistical methods | Y | Y |
|  |  | Additional statistical methods | N | N |
|  |  | Input of missing data | N | N |
|  |  | Software used | N | N |
| Results: flow | 12 | Participants flow | Y | Y |
|  |  | Enrollment | Y | Y |
|  |  | Assignment | Y | Y |
|  |  | Allocation and exposure | Y | Y |
|  |  | Follow-up | Y | Y |
|  |  | Analysis | Y | Y |
|  |  | Protocol deviations | N | N |
| Results: recruitment | 13 | Dates | N | N |
| Results: baseline | 14 | Baseline demographic | Y | Y |
|  |  | Baseline characteristics | Y | Y |
|  |  | Baseline comparison of those lost to follow-up and those retained | N/A | N/A |
|  |  | Comparison at baseline | Y | Y |
| Results: baseline equivalence | 15 | Equivalence | N/A | Y |
| Results: number analyzed | 16 | Number of participants | Y | Y |
|  |  | Intention to treat | Y | Y |
| Results: outcomes | 17 | Summary of results with effect size | N | N |
|  |  | Inclusion of null and negative findings | Y | Y |
|  |  | Pre-specified causal pathways | N/A | N/A |
| Results: ancillary analyses | 18 | Other analyses | N/A | N/A |
| Results: adverse events | 19 | Important adverse effects | N | N |
| Discussion | 20 | Interpretation | Y | Y |
|  |  | Mechanisms | Y | Y |
|  |  | Success and barriers | Y | Y |
|  |  | Discussion of research, programmatic, or policy implication | N | N |
| Discussion: generalizability | 21 | External validity | Y | Y |
| Discussion: overall evidence | 22 | General interpretation | Y | Y |
| **Total** |  |  | 33/58 | 38/58 |
