# Supplementary material for: Whole-body vibration training in obese subjects: A systematic review
Source: PLoS One. 2018 Sep 5;13(9):e0202866. doi: 10.1371/journal.pone.0202866 (PMC6124767; doi:10.1371/journal.pone.0202866)
Supplement: S3 Table — (DOCX) [file pone.0202866.s003.docx]

S3 Table: quality of reporting whole-body vibration treatment (Y: yes, N: no or not applicable).

| **Reference** | **Year** | **Brand, name, type** | **Vibration type** | **Vibration frequency** | **Peak-to-peak displacement** | **Peak or RMS acceleration** | **Accuracy of vibration settings** | **Feet skidding** | **Change of vibration settings** | **Rationale for vibration settings** | **Support devices** | **Footwear** | **Position or posture** | **Exercise performed** | **Supervision** | **TOT** |
| --- | --- | --- | --- | --- | --- | --- | --- | --- | --- | --- | --- | --- | --- | --- | --- | --- |
| [58] | 2010 | Y | Y | Y | N | N | N | N | Y | N | Y | N | N | Y | N | **6** |
| [56] | 2012 | Y | N | Y | Y | Y | N | N | Y | N | Y | N | Y | Y | Y | **9** |
| [60] | 2012 | Y | Y | Y | Y | N | N | N | Y | N | N | N | Y | Y | N | **7** |
| [51] | 2012 | Y | Y | Y | Y | N | N | N | Y | Y | N | N | Y | Y | Y | **9** |
| [61] | 2013 | Y | Y | Y | N | N | N | N | Y | N | N | N | Y | Y | Y | **7** |
| [62] | 2013 | Y | N | Y | Y | N | N | N | Y | N | N | N | Y | Y | Y | **7** |
| [53] | 2013 | Y | Y | Y | Y | N | N | N | N | N | N | N | N | Y | N | **5** |
| [57] | 2014 | Y | Y | Y | N | N | N | N | N | N | Y | N | N | Y | Y | **6** |
| [63] | 2014 | Y | Y | Y | Y | N | N | N | N | Y | N | N | Y | Y | Y | **8** |
| [26] | 2014 | Y | N | Y | Y | N | N | N | Y | Y | N | N | Y | Y | Y | **8** |
| [54] | 2014 | Y | N | Y | N | N | N | N | Y | N | N | N | Y | Y | Y | **6** |
| [48] | 2014 | Y | N | Y | Y | N | N | N | Y | N | N | N | Y | Y | N | **6** |
| [59] | 2015 | Y | N | Y | Y | N | N | N | Y | N | Y | N | Y | Y | Y | **8** |
| [50] | 2016 | Y | Y | Y | N | Y | N | N | Y | Y | N | Y | Y | Y | Y | **10** |
| [49] | 2016 | Y | N | Y | Y | N | N | N | Y | Y | N | N | Y | Y | Y | **8** |
| [52] | 2016 | Y | N | Y | Y | N | N | N | Y | Y | N | N | Y | Y | Y | **8** |
| [47] | 2017 | Y | Y | Y | Y | N | N | N | N | N | Y | N | Y | N | N | **6** |
| [55] | 2017 | Y | Y | Y | N | N | N | N | Y | Y | N | Y | Y | Y | Y | **9** |
|  | Tot | 18 | 10 | 18 | 12 | 2 | 0 | 0 | 14 | 7 | 5 | 2 | 15 | 17 | 13 |  |
